# The Need for Biomarkers in Diagnosis and Prognosis of Drug-Induced Liver Disease: Does Metabolomics Have Any Role?

**DOI:** 10.1155/2015/386186

**Published:** 2015-12-28

**Authors:** Paula Iruzubieta, Maria Teresa Arias-Loste, Lucía Barbier-Torres, Maria Luz Martinez-Chantar, Javier Crespo

**Affiliations:** ^1^Gastroenterology and Hepatology Department, Marqués de Valdecilla University Hospital, 39008 Santander, Spain; ^2^Infection, Immunity and Digestive Pathology Group, Research Institute Marqués de Valdecilla (IDIVAL), 39011 Santander, Spain; ^3^CIC bioGUNE, Metabolomics Unit, Centro de Investigación Biomédica en Red de Enfermedades Hepáticas y Digestivas (CIBERehd), 48160 Derio, Spain

## Abstract

Drug-induced liver injury (DILI) is a potentially fatal adverse event and the leading cause of acute liver failure in the US and in the majority of Europe. The liver can be affected directly, in a dose-dependent manner, or idiosyncratically, independently of the dose, and therefore unpredictably. Currently, DILI is a diagnosis of exclusion that physicians should suspect in patients with unexplained elevated liver enzymes. Therefore, new diagnostic and prognostic biomarkers are necessary to achieve an early and reliable diagnosis of DILI and thus improve the prognosis. Although several DILI biomarkers have been found through analytical and genetic tests and pharmacokinetic approaches, none of them have been able to display enough specificity and sensitivity, so new approaches are needed. In this sense, metabolomics is a strongly and promising emerging field that, from biofluids collected through minimally invasive procedures, can obtain early biomarkers of toxicity, which may constitute specific indicators of liver damage.

## 1. Introduction

Liver injury due to both prescription and over-the-counter drugs is a growing public health problem. Although drug-induced liver injury (DILI) is a rare cause of acute liver injury in clinical practice, it is the leading cause of acute liver failure (ALF) in the US and most of Europe. The estimated incidence is 10–15 cases/100,000 patient years [1, 2]. Acetaminophen (APAP) is the drug most frequently involved in DILI, representing over 50% of cases of ALF in adults in US [2, 3]. Moreover, a small proportion of patients may develop chronic liver disease [4–8]. Probably, the actual incidence of DILI is higher than thought, due in part to misdiagnosis as it is frequently difficult to identify. In most cases, liver injury is a consequence of mitochondrial damage leading to hepatocyte death. However, in different circumstances, the target of drug toxicity can be cholangiocytes or endothelial cells, causing cholestasis or sinusoidal obstruction syndrome instead.

DILI is usually categorized into “intrinsic” and “idiosyncratic.” The main intrinsic hepatotoxic drug is APAP, which is characterized by dose-dependent hepatotoxicity. In contrast, idiosyncratic DILI is not clearly related to drug dose, route, or duration of administration. Despite the extensive safety tests performed in the process of getting a drug to market, DILI remains enigmatic and cannot be predicted in preclinical and clinical trials. Even though different genetic variants and biomarkers have been associated with the risk of developing DILI, hepatotoxicity remains a very common side effect. These variants include different HLA alleles [9–11] and various serum biomarkers, such as miRNA-122, high-mobility group box-1 (HMGB-1), full length and caspase-cleaved keratin-18 (K-18), and glutamate dehydrogenase (GLDH) [12].

Since DILI is associated with increased morbidity and can lead to ALF, liver transplant, and death, it would be desirable that physicians could easily and early establish the diagnosis of DILI identifying those patients with a poor prognosis. This way, strategies to manage complications and improve prognosis of DILI could be designed more efficiently.

In this review we will focus on the potential role of metabolomic study on the diagnosis and prognosis of DILI.

## 2. The Main Challenges in DILI: Accuracy of Diagnosis and Prediction of Evolution

Currently, DILI is diagnosed by exclusion of other liver diseases and is frequently misdiagnosed. An important part in the diagnosis of DILI is to establish a temporal association between a drug and the onset of liver damage. However, in many cases, setting a causal link can be a complex task as most of the idiosyncratic drug-induced reactions occur roughly between a range of 1-2 weeks and 2-3 months from the onset of drug administration [13]. Moreover, a significant time lag between drug discontinuation and the appearance of elevated liver enzymes may exist [14, 15]. The indication for a liver biopsy in patients with suspected DILI is controversial due largely to the absence of pathognomonic histological findings [16]. In the case of liver damage mediated by APAP, blood concentration of APAP can be obtained early in the course of the reaction, which can confirm toxicity. However, APAP drug levels are not reliable predictors of liver damage evolution. Moreover, false positive concentrations of APAP have been observed in patients with ALF, especially in patients with high bilirubin levels [17]. Therefore, given the heterogeneity in terms of clinical presentation, biochemistry, and histology of DILI, current diagnosis is based on circumstantial evidence.

Importantly, our knowledge is scarce not only in terms of diagnostic accuracy. The natural history of DILI is still not completely understood. It is relatively common to find minimally symptomatic or asymptomatic elevated liver enzymes related to drug administration, which in most cases is solved by discontinuing the drug. Notably, in other cases it is not even necessary to discontinue the drug to achieve a complete and spontaneous remission of the side effect. This phenomenon of “adaptation” has even been observed in drugs with potential to cause ALF [18–20]. It is believed that severe liver damage occurs in a subset of patients with elevated liver enzymes that are unable to adapt to the initial mild toxicity. Currently there is no method able to distinguish between patients with benign and reversible elevation of liver enzymes from those in whom liver damage will progress. To improve both the diagnosis and the prognosis of DILI, it would be important to have early specific biomarkers available, which may enable physicians to improve the prognosis of DILI and, importantly, they could avoid unnecessary drug discontinuations in those cases in which no significant liver damage will develop. In this field, metabolomics is emerging forcefully in order to identify early toxicity biomarkers from biofluids collected through minimally invasive procedures that are specific indicators of liver damage.

## 3. Metabolomics: Concept and Definition

Since the early nineties, metabolomics has emerged strongly from the omics sciences [21, 22]. This science studies the set of metabolites (global metabolic profiling) present in a biological system, particularly in biofluids (serum, urine, feces, sweet, tears, and saliva). It is defined as a quantitative and multiparametric measurement of the response of a living organism to pathophysiologic stimulus or genetic modification [23]. One of the main objectives in metabolomics research is the discovery of specific metabolic profiles associated with the disease or the response to specific treatments.

Metabolomics offers a view distinct from Genomics and Proteomics. While Genomics and Proteomics tell us what “can happen,” metabolomics tells us what is “really happening” and, therefore, is the science that can better characterize the phenotypes of living organisms.

The main analytical methods used in metabolomic analyses are nuclear magnetic resonance (NMR) and mass spectrometry (MS). The NMR enables the measurement of many metabolites reliably and repeatedly, starting from conditioning process simple samples with a very high level of automation. MS is usually coupled with a separation technique such as liquid chromatography (LC) or gas chromatography (GC). LC-MS and GC-MS are able to analyze a large number of metabolites with high sensitivity (higher than the NMR) but treatment of samples is tedious. In order to analyze metabolomic data, many mathematical or statistical tools are required. The main pattern recognition technique used is principal component analysis (PCA), which is capable of classifying sample groups based on the inherent similarity or dissimilarity of their corresponding biochemical compositions [24].

Since the liver is the primary organ of metabolism and synthesizes the majority of the endogenous metabolites, it seems reasonable to assume that the effects of a drug causing DILI may be reflected in these endogenous metabolites. In this way, global metabolic profiling offers us the opportunity to identify biomarkers or patterns of biomarker changes related to drug toxicity in biofluid samples. To understand the results of metabolomic profiles, the key aspects of the pathophysiology of DILI and metabolites involved need to be well defined.

## 4. Pathophysiology of DILI

As mentioned above, DILI can be intrinsic to the drug, as produced by APAP, or idiosyncratic. Although both types seem to develop by different mechanisms, drugs with well-documented idiosyncratic DILI have been shown to have a dose-dependent component [25–27], suggesting an important role of reactive metabolites in the pathogenesis of idiosyncratic DILI as it is for APAP. On the other hand, there have been reports on APAP-induced liver injury after taking therapeutic doses suggesting idiosyncratic liver injury also by APAP [28–30].

Pathogenesis of APAP-induced liver injury is well known because its study in animal models is possible and reproducible, allowing us to understand, at least in part, the biological pathways leading to liver damage. Conversely, drugs that produce idiosyncratic DILI in humans rarely cause liver damage in experimental animals, probably because some baseline factors needed in the individual that enhances the damage, such as old age or the presence of infections, are not present in the model. For this reason, we have developed two animal models for studying this type of DILI: lipopolysaccharide (LPS) Costimulation, consisting in the administration of potentially hepatotoxic drug after nontoxic doses of LPS (bacteria endotoxin that promotes inflammation) [31] and Mn-SOD (+/−) heterozygous mice, which possess a decreased activity of the superoxide dismutase 2 (SOD2, the mitochondrial form of superoxide dismutase which protects against reactive oxygen species (ROS)) [32, 33]. Both models are aimed at creating added risk factors for drug administration, yet the underlying mechanisms involved in idiosyncratic DILI are not completely known. A model has been suggested in three stages. First, the drugs or their metabolites can cause direct cellular stress (intrinsic pathway), trigger immune reactions (extrinsic pathway), and/or directly alter mitochondrial function. After the first insult, the mitochondrial permeability transition (MPT) is produced, which ultimately leads to the onset of apoptosis or cell necrosis [34].

**Figure 1:**
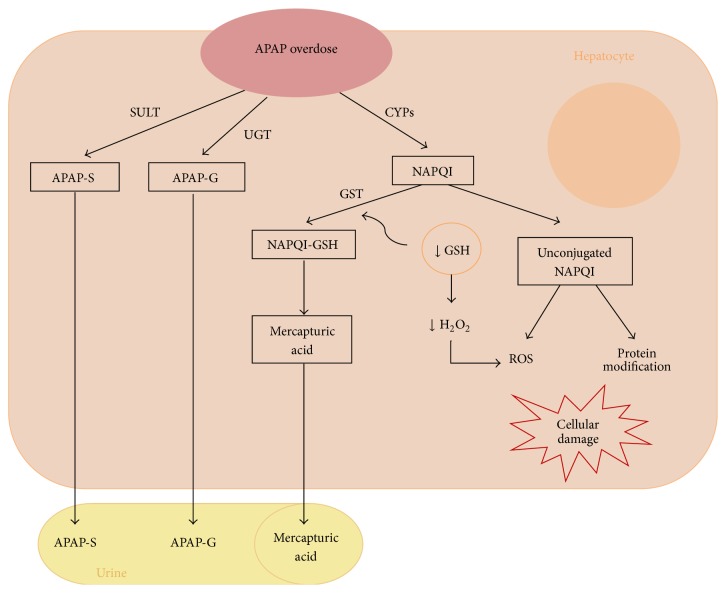
Hepatotoxicity of APAP. APAP: acetaminophen; SULT: sulfotransferase; UGT: glucuronosyltransferase; CYPs: P450 cytochromes; APAP-S: APAP-sulfonate; APAP-G: APAP-glucuronide; NAPQI: N-acetyl-p-benzoquinone imine; GST: glutathione S-transferase; GSH: glutathione; ROS: reactive oxygen species.

In the case of APAP, its pathogenesis is known largely thanks to metabolomics studies [35, 36]. APAP is predominantly metabolized in the liver by the conjugation reactions of sulfation and glucuronidation (phase II) leading to nontoxic sulfate and glucuronide conjugates, which are inert and are excreted in the urine. Only a small proportion of the drug administered at therapeutic doses is metabolized by several P450 cytochromes (CYPs) (phase I), specially CYP2E1, leading to N-acetyl-p-benzoquinone imine (NAPQI), a highly reactive intermediate metabolite, which is conjugated to glutathione (GSH) and detoxified to mercapturic acid [37]. When doses of APAP are excessive, phase II is saturated and the rapid generation of NAPQI can lead to depletion of cytoplasmic and mitochondrial glutathione [38]. Unconjugated NAPQI covalently binds to proteins and subcellular structures, which can potentially affect its functions [39] (Figure 1). Both GSH depletion (which is important in detoxifying H_2_O_2_ in the mitochondrial matrix and cytoplasm) and NAPQI covalent bonds can alter the electron transport chain, increasing the production of reactive oxygen species (ROS) [40, 41]. The ROS generated and NAPQI itself can oxidize and modify proteins, lipids, DNA, and other macromolecules, impairing their functions; thus a wide range of signaling pathways can be activated or inhibited [42]. Among these are crucial oxidation of proteins in the inner mitochondrial membrane by inducing MPT, which dissipates the proton gradient required for oxidative phosphorylation [43, 44], and the activation of JNK, which promotes cell damage and death by inducing MPT. This way, apoptotic factors, such as cytochrome c, which ultimately leads to hepatocyte death, are released [45, 46]. In addition, the APAP produces metabolic changes in the liver, such as rapid depletion of glycogen, probably an increased utilization of energy for repair and detoxification and an increase in triacylglycerol synthesis suggesting a slight increase in fatty liver after APAP [47]. All these changes and others, such as changes in small molecule metabolites that act as precursors or compounds of substances involved in the metabolism of drugs (e.g., glycine and S-adenosylmethionine (SAMe) important in the synthesis of GSH), can be evaluated by metabolomic tools allowing us to identify biomarkers of DILI from small single aliquots.

## 5. The Potential Role of Metabolic Profiling in the Diagnosis and Prognosis of DILI

**Figure 2:**
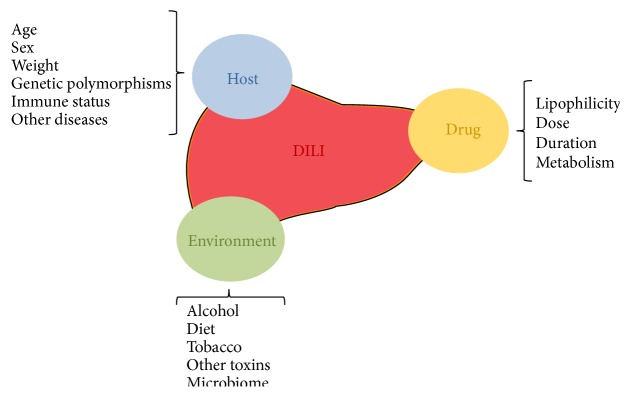
Factors which influence susceptibility to DILI.

Biomarkers are analyzed from blood, urine, or other biological samples that may provide insight into the severity, cause, or outcome of an injury to a specific tissue. In the case of DILI, biomarkers can improve the speed and accuracy of diagnosis and know the prognosis [21, 48]. For this purpose, various serum biomarkers have been proposed, such as alanine aminotransferase (ALT), sorbitol dehydrogenase (SDH), glutathione S-transferase (GST*α*), GLDH, microRNAs, CK-18, and HMGB-1, but these are not specific of DILI or liver disease [48]. Measuring derived products of a drug metabolism could be used to detect toxic responses to a drug, but these products may not be detectable in blood because they have very short half-lives or covalent binding protein may render them undetectable by standard pharmacokinetic analyses [49]. Further, various models have been developed based on laboratory test (Hy's Law, DrILTox ALF Score) to predict the risk of developing fulminant hepatic failure in patients with DILI [50–52] but without sufficient specificity and sensitivity. From a genetic standpoint, biomarkers of DILI have also been developed. They have demonstrated association of genetic variants, especially HLA alleles, with the risk of developing DILI [9–11, 53]. The problem with these biomarkers is that not only do genetic factors influence susceptibility to DILI [54] (Figure 2) but also environmental (concomitant disease), host-related (age, gender, and ethnicity), and drug-related factors (dose, metabolism, and lipophilicity) may modulate susceptibility to DILI [22, 54].

Therefore, pharmacokinetic approaches and genetic or analytical tests are not sufficient to diagnose and predict DILI, so new approaches are needed. Metabolomics, whose aim is to globally assess all the metabolites present in a biological sample, may represent an alternative in the search of specific markers of DILI. On one hand, the study of metabolomic profiles related to liver toxicity is particularly focused on the identification of preclinical susceptibility to hepatotoxicity by the assessment of specific patterns of endogenous metabolites, prior to the administration of a drug [40, 55]. This type of diagnostic approach has been called “pharmacometabonomic” approach. The potential of metabolomics to predict DILI was first suggested by Claytone et al. [55]. In this study, they found a correlation between NMR-based metabolomics profiles of urine samples of rats before administration of single toxic-threshold dose of APAP with the postdose histopathological damage. Translating metabolomics from animals to humans is complex, as a result of the great genetic heterogeneity and variations in environmental factors, such as age, nutritional status, intestinal microbiota, and lifestyle, which influences the composition of the metabolome [41–43]. Several studies have demonstrated that humans are characterized by individual metabolic phenotypes or “metabotypes” [56–58]; that is, there are areas of the metabolome, which remain stable over time and are characteristic of each person. Therefore, different metabotypes can predict differences in drug metabolism and susceptibility to DILI in different subjects. In this regard, several studies have evaluated the application of pharmacometabonomics to humans advocating an individualized drug therapy [59, 60]. Winnike et al. [44] analyzed the metabolomic profiles in urine samples from 58 healthy adults before and after receiving 4 g of APAP per day for 7 days (a regimen that produces mild liver injury in about one-third of subjects). Urine metabolomic profiles obtained 2 days prior to treatment were not sufficient to predict the development of mild liver damage, but the profiles obtained after a short period of administration of APAP were able to predict it. These results suggest that, before the drug administration, the differences in metabotypes are too small to be detected beyond the inherent variation of the population and that, after administration of the drug, changes in the endogenous metabolites can allow us to distinguish between those individuals who will adapt to the initial mild liver damage from those susceptible to developing a more severe liver damage [45].

Therefore, it could be assumed that the importance of the metabolomic analysis in the diagnosis and prognosis of DILI is due to the multifactorial nature of DILI, the lack of detectable changes in the overall pretreatment metabolites, and the absence of cost-effectiveness of implementing any DILI predictor test before starting a potentially hepatotoxic drug. In this regard, numerous studies in animal models have identified changes in the metabolomic profiles in the presence of liver damage due to different drugs [36, 46, 48–54, 56–69]. Moreover, in recent years several studies in both animal models and* in vitro *models have been published [70–85]. Nevertheless, it should be pointed out that these studies were mainly designed to help the pharmaceutical industry and drug agencies to identify potentially hepatotoxic drugs and to screen hepatotoxicity (before the elevation of liver enzymes). There are few human studies evaluating the use of metabolomics to discover biomarkers of DILI [86–88]. Kim et al. [86] analysed plasma and urinary samples from subjects treated with APAP (3 g/d for 7 days) using a NMR platform. Although all biochemical parameters of hepatic function were within normal range, the metabolomics data showed evident changes in urinary and plasma metabolites, identifying 14 and 10 endogenous metabolites in the urine and plasma, respectively, related to APAP treatment and possible changes in hepatic function. They concluded that urinary and plasmatic metabolic profile could be useful for the prediction of hepatotoxicity in human. Bhattacharyya et al. [87] described different metabolomic profiles regarding serum acylcarnitines, intermediates in the mitochondrial *β*-oxidation of fatty acids, in children in different settings: with no exposure to APAP, under therapeutic doses of APAP, and after APAP overdose. Levels of palmitoyl- and oleoylcarnitine were elevated in children both after therapeutic doses and after APAP overdose. Moreover, children with higher levels of acylcarnitines were those after APAP overdose. However, most of the serum samples in the group of children with overdoses of APAP were taken after starting NAC, and, furthermore, this analysis did not take into account the levels of liver enzymes and, therefore, it cannot ensure that high levels of acylcarnitines are indicative of APAP-induced liver injury. Recently, Huo and collaborators have published a study in humans, using a metabolomic approach to identify diagnostic biomarkers of DILI [88]. They evaluated the liver toxicity of sodium valproate using ultra-performance LC-MS and HNMR-based metabolomics analysis of serum samples from 34 epileptic patients receiving this drug. They found differences in metabolites involved in glycolysis, lipid metabolism, energy metabolism, and amino acids metabolism between patients with normal liver function and those with elevated liver enzymes due to the mentioned drug. Thus, they could define metabolites associated with valproate sodium-induced hepatotoxicity. This work demonstrates the potential of using metabolomics to discover biomarkers of hepatotoxicity.

## 6. Conclusions

One of the major concerns a clinician has to face when suspecting DILI is to establish an early and precise diagnosis in order to attempt to identify those patients at higher risk of significant liver damage and ALF. Only when this could be reached, measures to improve prognosis can be adopted. In this sense, the tremendous growth metabolomics has experienced over the last decade is notable, with remarkable applications in the area of liver toxicity. It is in this field where metabolomics has shown the potential of combining physiological and metabolic pathway information to drug toxicity studies. Moreover, its ability to rapidly detect many metabolites using NMR and MS from biofluid obtained by minimally invasive techniques makes metabolomics a promising tool in the discovery of biomarkers allocated to establish an early diagnosis and prognosis of DILI.

There are few human studies that use a metabolomic approach. Therefore, further studies are necessary to contrast different metabolomic profiles: first, comparing patients exposed to a drug with no liver damage to patients taking the same drug but developing DILI; second, comparing DILI patients to patients with liver disease of a different etiology.
